# Supplementary material for: Novel carbon skeletons activate human NicotinAMide Phosphoribosyl Transferase (NAMPT) enzyme in biochemical assay
Source: PLoS One. 2023 Mar 30;18(3):e0283428. doi: 10.1371/journal.pone.0283428 (PMC10062609; doi:10.1371/journal.pone.0283428)
Supplement: Attachment — Submitted filename: PONE-D-22-30462_review.docx [file pone.0283428.s002.docx]

The manuscript titled “Novel carbon skeletons activate human NicotinAMide PhosphoribosylTransferase (NAMPT) enzyme in biochemical assay” by Almeida et al describes the results of a docking-based virtual screening coupled with biochemical assay aimed at finding novel compounds activating NAMPT, which is the rate-limiting enzyme in the NAD^+^ salvage pathway, regulates the NAD^+^ biosynthetic capacity of cells.

The concerns regarding the manuscript are as follows:

- It is not clear which the NCI Diversity set was used for screening? III or IV? Is the screening library derived from only natural products? The authors should discuss that the both of virtual screening hits are polyphenolic compound?
- Is there any information about the fluorescence intensity of the compounds (2 and 20) themselves? If not, should be checked.
- Although the studies on NAT revealed the critical role of K189 in boosting NAMPT activity, the binding modes of other activators with diverse scaffolds should also be considered and docking studies should be performed focusing on additional important residues (https://doi.org/10.1016/j.apsb.2022.07.016).
- Since the importance of water-mediated h-bonds in the interaction for both inhibitors and activators is known, water molecules in active site should be kept during docking simulations.
- The NAMPT activators reported to date should be added to the screening library.
- The proposed binding modes of compounds 2 and 20 did not present the binding site properly. The figures need to be improved.
- The MD simulations should be performed for 2:NAMPT and 20:NAMPT complexes which would be support the findings.
- Please check the words throughout the manuscrip, fig or Fig?
